# A Successful Case of Immediate Transfusion

**Published:** 1872-10

**Authors:** J. H. Aveling

**Affiliations:** Physician to the Chelsea Hospital for Women


### A Successful Case of Immediate Transfusion.

By J. H. Aveling, M. D., Physician to the Chelsea Hospital for Women.

The following satisfactory case of transfusion proves that the old "immediate" method of performing the operation has many advantages—so many, indeed, as to lead to the belief that it must ere long be adopted by the profession. For one hundred and fifty years no other plan was known; and it is only during the last half century that "mediate" transfusion has been in vogue. The delay and difficulty in operating and the deteriorated condition of the blood transmitted are the great objections to this latter method. If it can be shown that the immediate plan is as successful as it is simple, transfusion will assume its proper place among the remedies of the healing art, and will be used both by physician and surgeon in all cases where deterioration or loss of blood threatens extinction of life.

Mr. F. E. Webb, of Maida-vale, has kindly furnished the following notes of this case previous to the time of my being called in :

"Mrs. W——, a small, fair lady, aged twenty-one, of rather delicate constitution, was seized with abdominal pains on the evening of March 24th. Early on the morning of the 25th I was sent for. The pains were frequent, but there were no expulsive efforts.. The os was slightly dilated and the vaginal secretion abundant. Labor proceeded slowly, owing to the brim of the pelvis being narrow; and the child's head did not descend into the cavity of the pelvis until one o'clock, when sharp expulsive efforts commenced. At two o'clock I began to give her chloroform at intervals, but not to insensibility at any time, until the child was born at 3:30 P. M. As soon as the child was detached smart hæmorrhage set in. I sent for ice, gave ergot and brandy freely, and, grasping the uterus, excited it to vigorous action. The hæmorrhage continuing, I found it necessary to detach the remaining half of the placenta, which was unusually adherent to what felt like the partially inverted fundus of the uterus. This I endoavored to replace, but without success, as I was obliged to desist my efforts at the time lest the shock should entirely extinguish life. Blood continued to flow freely, and the patient became rapidly exhausted and faint, and no pulse could be felt at the wrist. Ice in the vagina at length checked the bleeding; but, as there seemed every probability of the patient dying, the husband was requested to go for further advice. Dr. Cheadle came first, and shortly after Dr. Meadows, At this time (4:30 P. M.) there was no great amount of hæmorrhage, but we all agreed that the only prospect of saving the patient's life was to transfuse some blood into her viens. Dr. Cheadle kindly went for Dr. Aveling, knowing that he would probably have the necessary apparatus, and would be ready to perform the operation."

I found the patient in a most dangerous state of exhaustion, insensible, and no pulse to be found in either the temporal or radial arteries. The pupils were dilated, and did not contract when a light was placed near them. The hands and feet were cold, and the lips and face blanched. The heart's action was weak, and growing perceptibly more feeble. It was evident there was no time to be lost. A fold of skin at the bend of the patient's arm was raised, transfixed, and divided; when a large flattened blue vein became visible. This was opened, and the afferent tube with some difficulty, on account of the insufficient light of two candles, was adjusted. The arm of a coachman in the employ of the family was next prepared as in ordinary bleeding, and an incision made directly into the vein sufficiently large to admit the afferent tube. The man was then seated in a chair beside the bed, and the india-rubber portion of the apparatus filled with water having been attached to the tubes, the process of transfusing commenced. After a few drahms had been transmitted, Dr. Meadows, who kindly took charge of the afferent tube, thought he felt the skin rising near the incision, and suggested that the tube was not in the vein, but in the cellular tissue beside it. This proved to be true, and the tube had to be taken out and inserted into the vein. Its collapsed condition and the want of light made this no easy task; but it was at length effected, and the transfusion then went on steadily and easily until more than sixty drachms of blood had been injected. As the operation proceeded, the pulse at the wrists became perceptible, the lips less blanched, and warmth returned to the hands. The patient also became conscious for a short time, and said she was "dying." The mental improvement was not as marked and rapid as I anticipated; but this was, perhaps, due to the quantity of brandy she had taken. In a few hours, however, she became quite conscious, spoke, took nourishment, and began her fresh lease of life. The wound in her arm healed by first intention; but it opened again a few days after to allow some pus to escape, the result of the accident already alluded to. When the patient was sufficiently recovered she was placed under the influence of chloroform, and the uterus, which had become completely inverted, was returned to its normal position. After this operation Mrs. W—— improved rapidly, and is now quite well. It would be ungrateful not to admit that a large part of the success of the operation is due to the able assistance I received from Dr. Meadows and Mr. Webb, and, I must add, from the coachman, who was not only collected and cheerful, but able to make several useful suggestions during the process of transfusion.—*London Lancet.*

